# Nonsteroidal Topical Immunomodulators in Allergology and Dermatology

**DOI:** 10.1155/2016/5185303

**Published:** 2016-04-06

**Authors:** Marina Jovanović, Zoran Golušin

**Affiliations:** Department of Allergy and Clinical Immunology, Clinic of Dermatovenereology Diseases, Clinical Center of Vojvodina, Faculty of Medicine, University of Novi Sad, 21 000 Novi Sad, Serbia

## Abstract

The purpose of this study was to review currently available literature data concerning pathomechanisms of action, indications, treatment efficacy, as well as side effects of nonsteroidal immunomodulators used in dermatology, primarily for the treatment of allergic dermatoses. MEDLINE search was undertaken using the key words “Topical Immunomodulators, Dermatology and Allergy”. Full articles, and nothing but full articles, were used.

## 1. Introduction

Topical immunomodulators are agents that regulate the local immune response of the skin. Being the largest immune organ, the skin is a target, where in physiological conditions antigen presentation and induction of immune response are in balance. Immunomodulators are indicated if these two immune processes are unbalanced. Compared with systemic immunomodulatory agents, topical immunomodulators show comparable efficacy, ease of application, and greater safety for longer periods without aggressive monitoring. In regard to their mechanism of action, they are either immunostimulatory or immunosuppressive. They are classified into steroidal and nonsteroidal immunomodulatory agents, but generally nonsteroidal topical immunomodulators include the following agents: macrolactams, contact sensitizers, immunostimulants, and miscellaneous agents [1].

## 2. Macrolactam Immunomodulators

### 2.1. Tacrolimus (FK 506)

The word* tacrolimus* stands for Tsukuba—a geographical region in Japan after which the fungus* Streptomyces tsukubaensis* was named and from which* tacrolimus* was isolated [2]. It penetrates the cutaneous barrier to a much greater extent than cyclosporine but is not metabolized locally in the skin. It is only minimally absorbed, with 0.5% of the locally applied drug detected in blood, which is undetectable or subtherapeutic [1]. After binding to cytoplasmic immunophilins (known as tacrolimus-binding proteins), tacrolimus acts by selective inhibition of the phosphatase activity of calcineurin, leading to reduced dephosphorylation of the nuclear factor of activated T-cells, inhibiting its translocation into the nucleus, and thus preventing the transcription of several cytokines, including interleukin-2 (IL-2) genes and interferon-*γ* (IFN-*γ*) in T-cells [3]. Topical calcineurin inhibitors (TCIs) have been shown to have immunomodulatory effects by inhibiting activation/maturation of T-cells; production of cytokines IL-2, -4, -5, tumor necrosis factor-*α* (TNF-*α*), IFN-*γ*, granulocyte-macrophage colony-stimulating factor (GM-CSF); expression of IL-8 receptors on keratinocytes; decreasing the expression of high-affinity receptor for immunoglobulin E class (Fc*ε*RI) and CD36 molecules on Langerhans cells (LC) [4]; inhibiting mast cells degranulation [5]; reducing the number of LC and inflammatory dendritic epidermal cells (IDEC) and decreasing the expression of chemokine receptor CCR7 [3]. Tacrolimus restores the epidermal barrier and thus inhibits bronchial hyperactivity and reduces production of immunoglobulins class E (IgE) [6].

Tacrolimus is used in the treatment of atopic dermatitis (AD) (the only one approved by the Food and Drug Administration Agency (FDA)), but it has been effectively used to treat psoriasis, pyoderma gangrenosum, lichen planus, graft versus host disease (GVHD), allergic contact dermatitis, rosacea, discoid lupus erythematosus, erythema in a systemic lupus erythematosus (SLE) and dermatomyositis, chronic actinic dermatitis (CAD) [3], allergic asthma, allergic rhinitis and conjunctivitis, vitiligo, and venous ulcerations in rheumatoid arthritis [1]. In children with AD, 0.03% tacrolimus ointment is used twice a day, and 0.1% ointment is used twice a day in adults during a one-year period of treatment. The efficacy of tacrolimus is comparable to that of the potent topical corticosteroids, for example, 0.12% betamethasone valerate ointment [7] or mometasone furoate 0.1% cream (a mid-potent according to the American Contact Dermatitis Society classification) [8]. Tacrolimus ointment does not decrease collagen synthesis of the skin; this is one of its the main advantages compared to topical corticosteroids [7]. Lower efficiency in the treatment of dermatoses with an intact epidermal barrier function can be explained by harder penetration of tacrolimus due to its molecular weight (822 daltons) which is approximately the same as pimecrolimus (811 daltons), but significantly higher than the upper limit necessary for penetration into the skin (500 daltons) [9]. However, recent studies reported a novel modified nanolipid carrier system for topical delivery of tacrolimus (T-MNLC) to be more appealing and beneficial to the patients with better compliance to treating large skin areas of AD requiring long-term treatment [10].

Tacrolimus has shown encouraging results in the treatment of inverse psoriasis and psoriatic lesions on the face [11].

In the treatment of mucosal erosive lichen planus, tacrolimus has shown a positive therapeutic response [12].

In the management of pyoderma gangrenosum (PG), 0.3% tacrolimus formulated in carmellose sodium paste in combination with 0.05% clobetasol propionate ointment; tacrolimus demonstrated good therapeutic efficacy [13] by suppressing neutrophil chemotaxis: suppression of GM-CSF and IL-8. Therapy with topical tacrolimus 0.1% ointment twice per day resulted in a significant removal of pustular penile PG after 7 days of treatment [14]. In the therapy of localized pyoderma gangrenosum (PGL), 0.1% tacrolimus ointment (2x per day till resolution, then 1x a day for 3 months, and 2x per week for 6–12 months) is the first-line therapy. PGL has been defined by the following criteria: ≤5% of the skin surface affected, ≤3 lesions, short duration, negative microbiological test results, and unknown etiology [15].

Topical 0.1% tacrolimus ointment with photochemotherapy is suitable adjuvant therapy in the management of erythema and itching in GVHD [16].

Topical 0.075% tacrolimus ointment shows beneficial effects in the treatment of corticosteroid-induced rosacea, by inhibition of IL-1*α* [17].

In the treatment of resistant cutaneous lupus erythematosus in adults, the use of 0.3% tacrolimus in combination with 0.05% clobetasol propionate ointment, during 1 month to 6 years, showed few side effects (suppression of glucocorticoid-induced IL-1*α*), but a significantly greater anti-inflammatory effect than with 0.1% tacrolimus monotherapy [18].

In chronic actinic dermatitis (CAD), tacrolimus decreases the number of LC and DEC, as well as expression of CCR7 with the impeding of antigen presentation and dendritic cells (DC) homing, thus interfering with the induction of delayed-type hypersensitivity reaction (DTH) [3].

In allergic asthma (AS), allergic rhinitis (AR), and allergic conjunctivitis (AC), tacrolimus restores epidermal barrier which results in the reduction of bronchial hyperactivity and IgE production [6]. Tacrolimus is currently available for the topical treatment of severe allergic conjunctivitis. In allergic conjunctivitis and blepharitis tacrolimus does not elevate intraocular pressure; thus it is safer than topical corticosteroids [19].

In vitiligo lesions, it has been shown that tacrolimus increases migration of melanocytes significantly more than ultraviolet UV radiation by the following actions: increase in the activity of metalloproteinases which is significantly higher than when induced by UV; some reduction of synthesis of TNF-*α* (TNF-*α* reducing the proliferation of melanocytes) [20]. In combination with a narrow-band UVB phototherapy (NB-UVB), tacrolimus showed synergistic effects [21–23]. In the treatment of vitiligo, tacrolimus is an alternative to corticosteroids (faster repigmentation and absence of atrophy) in children and in lesions on the face, neck, or flexural areas [24, 25].

Wound healing in experimental animals has demonstrated that unlike topical corticosteroids (CS), which seemed to delay wound healing, tacrolimus exhibited no negative effects; it increased epithelization, proliferation of fibroblasts, collagen synthesis, and neutrophil polymorphonuclear leukocytes (PMN) infiltration [26].

Successful use of tacrolimus in the treatment of venous ulcerations without secondary infections in rheumatoid arthritis (RA) suggests a role for T-cells in rheumatoid ulcers. Topical tacrolimus inhibits cytokine production and early activation of T-cells; thus it is locally immunomodulating, which could suppress the vasculitis component likely to be involved in ulcers associated with RA and thus promote healing. Another possible mechanism is increased collagen synthesis [27].

Treatment of eosinophilic pustulosis of infancy with topical 0.03% tacrolimus ointment twice daily (inhibition of Th-2 cytokines) is the first-line therapy [28]. Tacrolimus is safe in children under 2 years of age; a pharmacokinetic multicenter study has been done in <2-year-old children which shows no increased serum tacrolimus levels [29]. It has been shown that liposomal formulations of tacrolimus have a 9-fold increase in skin levels compared to the systemic agents [30].

Tacrolimus side effects include burning, erythema, headache, and secondary infections [1, 31, 32]. Topical calcineurin inhibitors (TCIs) were first introduced for the treatment of AD in 1997 [33], with the final beneficial safety concerns announced in 2006 [34].

### 2.2. Pimecrolimus (ASM-981)

Isolated from* Streptomyces hygroscopicus* var.* Ascomycetes*, pimecrolimus like tacrolimus is a calcineurin inhibitor which inhibits T-cell stimulation by antigen-presenting cells, blocking both T helper cell 1 (Th1) cytokines such as IL-2 and interferon (IFN-*γ*) and T helper cell 2 (Th2) cytokines including IL-4 10 [1]. It also inhibits mast cell release of hexosaminidase, tryptase, and histamine [35]. A recent study done in an oxazolone-induced AD murine model has shown that topical pimecrolimus, like topical glucocorticoids, improved the AD-like skin lesions and barrier impairment (important for asthma development in AD patients), by suppressing thymic stromal lymphopoietin- (TSLP-) related allergic inflammation [36]. TSLP is an epithelial cell-derived IL-7-like cytokine which has an important role in allergic inflammatory immune response, particularly in dendritic cell-mediated allergic inflammation in AS and AD, since it converts human epidermal Langerhans cells into antigen-presenting cells which than induce proallergic T-cells.

Compared to tacrolimus, pimecrolimus shows lower skin penetration [37], higher affinity to epithelial structures, lower affinity to lymphoid structures, lower immunosuppressive effects [38], and threefold lower affinity to macrophilin-12 (FKBP12) [39].

Pimecrolimus is used in the treatment of AD (the only one indication approved by the FDA), but it has been effectively used to treat erosive oral and genital lichen planus, vulvar lichen sclerosus, Fox-Fordyce disease, intertriginous psoriasis, seborrheic dermatitis, erosive circinate balanitis, discoid lupus erythematosus, vulvar pruritus, vitiligo, and GVHD [40].

In the treatment of AD, 1% pimecrolimus cream, used twice a day, shows the highest efficacy but the similar local tolerability profile in comparison with its 0.05%, 0.2%, and 0.6% concentrations [41]. Pimecrolimus has similar efficacy as low to moderately potent topical corticosteroid creams for mild to moderate AD during the first 5 to 6 years of life, with similar rates of adverse events [42]. In the treatment of pediatric AD in a short-term, continuous-use course, pimecrolimus 1% cream versus tacrolimus 0.03% ointment has a similar efficiency as assessed by the improvement in the Investigator's Global Assessment (IGA) scale and is equally well tolerated [43].

Topical 1% pimecrolimus cream used in the treatment of moderate and severe AD in 1.133 children aged 3–23 months up to 2 years showed therapeutic efficacy without increased risk of percutaneous absorption, with no increased incidence of side effects compared to the vehicle alone, without increased incidence of noncutaneous infections compared to the vehicle alone, with no increase in the incidence of skin infections in relation to the vehicle, with no signs of immunosuppression after 2 years of therapy [44].

Nowadays, topical calcineurin inhibitors are primarily used in the treatment of AD, which is not defined as a genetically predisposed dry hypersensitive skin any more, but as acute eczematous skin, which emphasizes the crucial role in skin barrier integrity and extrinsic AD [45–47]. Topical therapy includes basic therapy that enhances the restoration and maintenance of the epidermal barrier (hydration, lipid substitution, especially ceramides, wet and occlusive dressings), anti-inflammatory and immunosuppressive therapy which involves application of topical CS, TICs (tacrolimus/pimecrolimus), and tar preparations. Topical anti-inflammatory therapy of the inflamed skin can be reactive, when CSs or calcineurin inhibitors are applied only on affected skin, and proactive, which is also prophylactic, when the drug is applied intermittently twice a week to those parts of the skin which had previously been affected with simultaneous use of emollients. TCIs tacrolimus/pimecrolimus have been approved by the FDA for the treatment of AD in individuals aged 2 years and over and in cases that do not require systemic therapy, or the disease showed to be resistant to topical corticosteroids.

**Figure 1:**
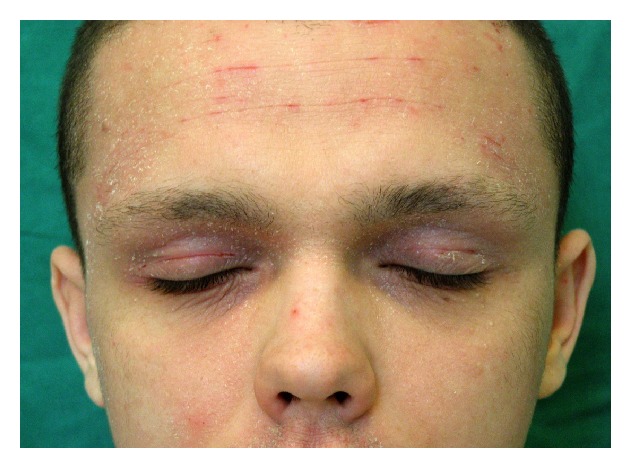
Atopic dermatitis on the face before treatment.

**Figure 2:**
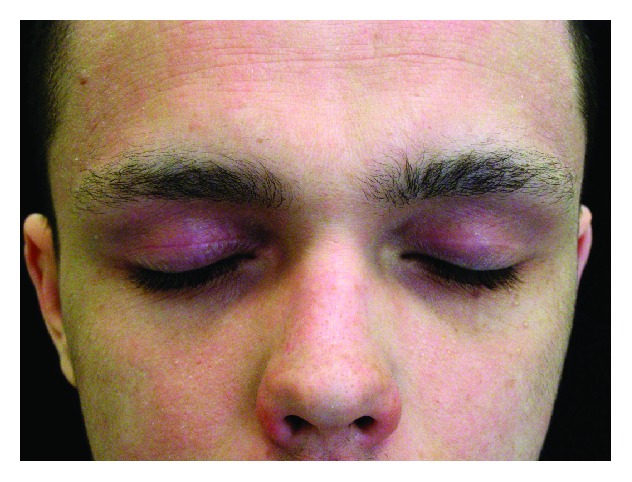
Atopic dermatitis on the face seven days after treatment with 1% pimecrolimus cream.

The main advantage of this therapy is long-term use at large joint folds, periorbital region, and face, without consequential atrophy, telangiectasia, and striae (Figures 1 and 2). In general, tacrolimus/pimecrolimus should not be used in combination with corticosteroids, whereas photoprotection is an integral part of treatment. Proactive use of pimecrolimus/tacrolimus prevents itching and relapses of mild to moderately severe forms of the disease [45, 48–50].

In February of 2005 (in agreement with topical corticosteroid phobia), FDA issued an alert warning about the possible risks of lymphoma and nonmelanoma skin cancer associated with TCIs, but quite soon, after considerable evaluations, the American Academy of Dermatology Association Task Force has announced that “there is no causal proof that TCIs cause lymphoma or non-melanoma skin cancer” [34]. Moreover, it was only a theoretical assumption that followed the appearance of tumors in experimental animals systemically exposed to high drug concentrations [51]. Aforementioned analyses revealed the following: during three years, 1.7 million people were treated with tacrolimus; 11 lymphomas were detected, none in children; 5 million people were treated with pimecrolimus; and 4 lymphomas, 1 basal cell carcinoma (BCC), and 1 squamous cell carcinoma (SCC) were detected [52, 53]. The investigation on the safety profile of TCIs regarding increased risk for the development of lymphoma was conducted retrospectively evaluating the association between topical immunosuppressants and lymphoma in a cohort study of patients with AD and revealed the following: two hundred and ninety-four cases of lymphoma occurred in 293.253 patients, 81 in patients younger than 20 years; the adjusted analysis yielded OR (95% CI) for the following: disease severity (OR 2.4; 95% CI 1.5–3.8), oral steroids (OR 1.5; CI 1.0–2.4), “superpotent” topical steroids (OR 1.2; 95% CI 0.8–1.8), “low potency” topical steroids (OR 1.1; 95% CI 0.7–1.6); pimecrolimus (OR 0.8; 95% CI 0.4–1.6); tacrolimus (OR 0.8; 95% CI 0.4–1.7); and concomitant topical steroids, pimecrolimus, and tacrolimus (OR 1.0; 95% CI 0.3–4.1). The study revealed no increased risk of lymphoma in patients treated with TCIs [54]. With photoprotection, TCIs have a favorable safety profile without evidence for increased risk for lymphoma [55]. Although there is no evidence of increased risk, clinicians should discuss the various risks with the patient or guardian and document discussion in detail [51].

Pimecrolimus showed a good safety profile in children >3 months of age [56–58].

A randomised, double-blind study, conducted in 10 patients using microplaque assay, 0.3% and 1% pimecrolimus ointments under occlusion for 2 weeks, showed a comparable efficacy to clobetasol-17-propionate ointment (0.05%) [59].

The therapeutic efficacy of pimecrolimus 1% cream in the treatment of genital lichen sclerosus has been demonstrated to be comparable with an ultrapotent CS, clobetasol-17-propionate 0.05% [60].

In the treatment of seborrheic dermatitis, 1% cream showed a favorable effect comparable with ketoconazole 2% cream [61] and betamethasone 17-valerate 0.1% cream [62].

### 2.3. Sirolimus (Rapamycin)

Isolated from* Streptomyces hygroscopicus* in Rapa Nui Island, sirolimus has great immunosuppressive effects building with macrophilin-12 (FKBP12). The target protein of this complex is serine-kinase, “mammalian target of rapamycin (mTOR)” that regulates cell growth. By inhibition of this protease, sirolimus inhibits the cytokine-dependent proliferation of T-cells. In relation to sirolimus, everolimus shows enhanced water solubility because of its additional hydroxy group.

Beneficial therapeutic effects of sirolimus were reported in the treatment of tuberous sclerosis complex (TSC), which results from mutations in a gene or genes that are part of a tumor suppression complex, involving the signal cascade pathway in which the mammalian target of rapamycin (mTOR) is mainly involved. In TSC, the inhibition of mTOR complex-1 is deactivated, leading to an upregulation of mTOR, causing uncontrolled cellular growth, proliferation, and protein synthesis [63]. When used in the treatment of children ≥36 weeks of age, applied once a day, 3 times per week for 9 months, it has shown efficacy in managing the symptoms of TSC through regression of facial angiofibromas [64]. The first case of successful treatment using topical everolimus was published in 2014 [65].

### 2.4. Cyclosporine

Cyclosporine is a lipophilic cyclic polypeptide isolated from* Tolypocladium inflatum gams*. Cyclosporine is a prodrug that becomes active only after forming a complex with an intracytoplasmic immunophilin (protein) known as cyclophilin. It showed therapeutic efficacy in cases of refractory standard topical corticosteroid therapy in the following diseases: oral pemphigus (5 mL of solution, 100 mg/mL, 5-minute mouthwash 3x/day, 1/day after 6 months); lichen planus (erosive, oral, and vulvar); pyoderma gangrenosum; and benign familial pemphigus [1]. Oral solution (100 mg/mL) has been shown effectiveness in the treatment of benign familial pemphigus compared to potent corticosteroids [66]. It does not penetrate the skin probably due to larger molecular size. Like tacrolimus, topical cyclosporine is currently available for topical treatment of severe allergic conjunctivitis [19].

## 3. Contact Sensitizers

The immunotherapeutic effectiveness of contact allergens in autoimmune diseases, primarily in alopecia areata (AA), was previously attributed solely to mechanisms of competitive inhibition, while today it is thought to be a consequence of immunomodulation: diphenylcyclopropenone (DPCP) affects T-cell activation and cytokine release [67]; squaric acid dibutyl ester (SADBE) exerts extravasation and recruitment of activated autoreactive T-cells [68].

### 3.1. Diphenylcyclopropenone

Being recognized as contact allergen with immunomodulating capacity, diphenylcyclopropenone (DPCP) has been widely accepted for the treatment of AA; moreover, it exhibited beneficial effects in patients with common warts as well as metastatic melanomas [1].

In AA, DPCP induces allergic response in 98-99% and a cosmetically acceptable hair regrowth in 28–80%. Its beneficial effects were in the past exclusively attributed to “competitive antigen inhibition.” Not strictly “contact allergic,” but also “immunomodulatory” effects [69] can be achieved due to the following DPCP actions: decreasing expression of human leukocyte antigens HLA-ABC and -DR antigens in the epithelium of lower hair follicles; impairing (hair) antigen presentation; and increasing the number of CD8+ lymphocytes (CD8+ Ly). Since CD8+ Ly are considered to be directly involved in hair destruction, their increased number may suggest that during DCP treatment they regain normal reactivity to hair antigens [70]. Generation of T-suppressor CD8 cells within the area treated with DPCP may exert a nonspecific inhibitory effect directed against the autoimmune reaction to an unidentified hair-associated autoantigen, with typical features of contact dermatitis with increased CD8 cells in the inflammatory infiltrate; it seems that DPCP acts by replacing T-cell subsets that have an epithelial cell growth inhibitory cytokine profile such as IFN-*γ* and transforming growth factor *β* (TGF-*β*), with delayed-type hypersensitivity T-cell subsets that have a stimulatory cytokine profile such as IL-2, IL-8, IL-10, and TNF-*α* [71]. It has also been shown that, in patients with AA, DPCP shows immunomodulatory effect such as increased expression of vascular endothelial growth factor (VEGF) in follicular keratinocytes and endothelial cells in the skin of the affected AA which stimulates angiogenesis and increases the expression of skin-associated chemokine CCL27 (CCL27) on keratinocytes, which stimulates the accumulation of cutaneous lymphocyte-associated antigen (CLA) + T-cell subsets to replace autoreactive (allergen specific) CD4 + T-cell subsets leading to a decrease in the CD4/CD8 ratio [67]. Regarding its side effects, tolerance is produced in 10–12% after 7–18 months (increasing CD4 + T-cell subsets in infiltrates); it is not recommended for children <15 years; contraceptives are mandatory (metabolites have a mutagenic potential).

It is assumed that the mechanism of action which induces positive response of DPCP in the treatment of common warts during 0.5–14 months on the feet (2% solution in acetone) and hands (0.1% solution in acetone) lies in the reduction in the number of CD4 + T-cells and increasing the CD4/CD8 ratio [1].

The induction of contact sensitization and then allergic contact dermatitis by 0.005%–0.01%–0.03% DPCP in aqueous cream showed therapeutic efficacy in the treatment of metastatic cutaneous melanoma without distant metastases, with exclusively cutaneous or local/recurrent or in-transit cutaneous metastases, and refractory melanomas unresponsive to conventional treatment methods. It is assumed that the mechanism of action is based on stimulation of Th17 cellular immune responses [72]. In order to achieve a moderate contact allergic response promptly, 0.01%−0.03% DPCP in aqueous cream was combined with 5% imiquimod cream 3x week: complete clearance of cutaneous disease was achieved in 46% and partial response in a further 38% of patients; mean DPCP treatment duration to clearance of all cutaneous lesions lasted 8 months (range 1–24); and mean duration of complete response was 17 months (range 1–78) [73]. Imiquimod has both immunomodulatory antiviral and antitumor Th1 effects, mediated via activation of Toll-like receptor 7 and 8 and increased production of IFN-*α*, IFN-*γ*, TNF-*α*, IL-1, IL-6, IL-8, IL-10, and IL-12 [73] and probably via enhanced inhibition of angiogenesis against micrometastases [74].

### 3.2. Dinitrochlorobenzene

Allergic contact dermatitis caused by dinitrochlorobenzene (DNCB) has been effectively used in the treatment of nonmelanoma skin cancers (NMSCs): Bowen disease, actinic keratosis, basal cell carcinoma,* human immunodeficiency virus* (HIV) infection (DNCB modulates Langerhans cell function, which plays an important role in HIV infection), and severe AD resistant to conventional therapy [75]. It has been suggested that the sensitizing agent in NMSC acts as a hapten and interacts with weak tumor antigens that by themselves are not sufficiently immunogenic to evoke an effective immune response [1].

DNCB contains contaminants that are mutagenic and carcinogenic to animals; when topically applied, more than 40% of the drug is absorbed systemically. DNCB and mechlorethamine have now largely been replaced by DPC and SADBE in the therapy of AA [76].

## 4. Immunostimulators

### 4.1. Imiquimod

Imiquimod, a synthetic immunomodulator, has been widely approved for the treatment of actinic keratosis (infiltration CD4+ T-cells), genital warts, and superficial BCC, but its topical application has also been effective in the treatment of common warts, keloids (locally increases IFN-*α* which than enhances keloidal collagenase activity), molluscum contagiosum infection, extramammary Paget's disease, and lentigo maligna [77].

Imiquimod enhances the innate immune response through binding to Toll-like receptor 7 in the monocytes and macrophages and increasing the production of cytokines: IFN-*α*, TNF-*α*, IL-1, IL-6, IL-8, IL-10, and IL-12. It also stimulates acquired immune response through the stimulation of B-cells, natural killer cells, and migration of LC [1]. Imiquimod 5% cream has immunoregulatory effects, local antitumor effects, and local antiviral effects. Since imiquimod indirectly stimulates Th-1 cells to release IFN-*γ*, which plays a role in cytotoxic T-cell killing of viral cells [1], it proved successful as adjuvant therapy in the treatment of genital herpes in immunocompromised individuals [78].

### 4.2. Resiquimod (R-848)

Imidazoquinoline amine 0.01% gel is a potent, soluble analogue of imiquimod. It showed effectiveness in the treatment of recurrent genital herpes by reducing the frequency of mucosal HSV-2 reactivation (genital shedding) [79], but not by reducing acute HSV-2 genital shedding [80].

## 5. Miscellaneous Agents

Miscellaneous topical immunomodulators include calcipotriol, calcitriol, tacalcitol, maxacalcitol, anthralin, zinc, and interferon-*α* (IFN-*α*, IFN-*α*2b) [1], while IFN-*α*2b, IFN-*β*1a, and intralesional bacillus Calmette-Guérin are predominantly intralesional recombinant immunomodulators.

### 5.1. Calcipotriol

Calcipotriol is a synthetic 1,25-dihydroxyvitamin D3 vitamin analogue which performs its immunomodulatory effects by binding to vitamin D receptors (VDR) present on the keratinocytes, thus inhibiting cellular proliferation and inducing cellular differentiation of these cells; it acts on monocytes, macrophages, and B and T lymphocytes to inhibit thymocyte proliferation induced by IL-1 and the release of IL-6 and IFN-*γ* from activated mononuclear cells; it also reduces the number of infiltrating neutrophils and causes progressive reduction in dermal cellular infiltrate with a shift from CD4+ helper cells to CD8+ suppressor cells [1]. The advantages of topical calcipotriol are as follows: it does not induce skin atrophy or photosensitization; it is well tolerated in children; and it is not teratogenic (elective abortion is not indicated). Its side effect is skin irritation, especially on the face, and it is not routinely recommended to patients with atopic dermatitis, neurodermatitis, or nummular eczema [1].

Calcipotriol (0.005% ointment, cream, and solution) showed favorable therapeutic effects in the treatment of plaque psoriasis (either alone or as an adjuvant to dithranol, topical steroids, and phototherapy, as twice daily application with maximum weekly application of 100 g in adults and 50 g in children); vitiligo (regulates melanin synthesis and restores calcium homeostasis in melanocytes/keratinocytes); keratinization disorders; and miscellaneous skin disorders [1].

Calcipotriol has good therapeutic effects in the treatment of vitiligo combined with NB-UVB therapy [81] or in combination with PUVA (psoralen and ultraviolet irradiation) phototherapy [82] that can be attributed to their synergistic effects. It is assumed that calcipotriol stimulates the expression of endothelin B and stem cell factor/c-kit cytokines, which results in activation of melanocytes and increased tyrosinase activity, and normalizes aberrant calcium homeostasis in keratinocytes and melanocytes present in vitiligo. Its immunomodulatory action is likely mediated by inhibiting the transition of T-cells from early to late G1 phase, which then decreases the synthesis of TNF-a and IFN-*γ* [81].

In regard to keratinization disorders, the literature data obtained from large control studies as well as case reports reported that calcipotriol has also demonstrated its usefulness in the treatment of keratinization disorders other than psoriasis, such as juvenile and adult-onset pityriasis rubra pilaris, inflammatory linear verrucous epidermal nevus, ichthyosis vulgaris, epidermolytic hyperkeratosis, lamellar ichthyosis, Sjögren-Larsson syndrome, ichthyosis linearis circumflexa, X-linked ichthyosis, hereditary palmoplantar keratoderma, acanthosis nigricans, keratosis pilaris, and Darier's disease [1].

### 5.2. Calcitriol

Hormonally active vitamin D3 [1a,25-dihydroxyvitamin D3, 1a,25(OH)2D3] is a metabolite, also called calcitriol, which is synthesized in keratinocytes. It is available as ointment (3 *μ*g/g); it is suitable for the treatment of psoriasis on the face, neck, and flexural areas, where it exhibits better efficacy than calcipotriol. In the treatment of psoriasis it may be used as monotherapy or as an adjuvant therapy with a topical corticosteroid or UVB phototherapy (the maximum recommended dose of calcitriol ointment should not exceed more than 200 g per week in adults and 100 g in children) [83]. In combination with corticosteroids, it reduces their side effects by restoring the following: epidermal permeability by increasing lipids (formation of lipid bodies and the activity of the epidermal lipid synthesis-related enzymes) and antimicrobial barrier (by increasing antimicrobial peptides) [84]. A concentration of 0.1 *μ*g/cm^2^ has a protective function of the UV-damaged skin DNA: it reduces thymine dimers by reducing nitric oxide products and enhances DNA repair by increasing postirradiation levels of p53. Higher doses (1 *μ*g/cm^2^) induce immunosuppression in the skin (reduced Mantoux response) due to increase of transforming growth factor *β* (TGF-*β*) in keratinocytes, which results in impaired migration of LC (Langerhans cells); inhibition of LC maturation; suppression of IL-12 production; and enhanced IL-10 production in dendritic cells, including LCs, which results in decreased T-cell activation [85]. Calcitriol is not for oral, ophthalmic, or intravaginal use, and it is not recommended for children younger than 15 years of age, as well as pregnant women [83].

### 5.3. Tacalcitol

Tacalcitol is a synthetic analogue of 1,25 dihydroxyvitamin D3, available as ointment (4 *μ*g/g); it is used for the treatment of psoriasis, and in combination with NB-UVB phototherapy, applied twice a week, it shows synergism in the treatment of generalized vitiligo by increasing c-Kit mRNA (receptor tyrosine kinase protein in humans encoded by the KIT gene) expression in irradiated melanocytes [86].

### 5.4. Maxacalcitol

A synthetic analogue of vitamin D3 (1 alpha, 25-dihydroxy-22-oxacalcitriol), maxacalcitol is available as ointment (0.0025%); it is used in the treatment of psoriasis as monotherapy, as an adjuvant therapy with NB-UVB phototherapy [87], or with cyclosporine A (Cys A), particularly in order to maintain remission [88].

### 5.5. Anthralin

Anthralin (1,8-dihydroxy-9-anthrone) is an immunomodulator which was previously thought to have only irritant properties in the treatment of AA; it exerts an antimitotic effect by inhibiting DNA synthesis, repair, and replication within keratinocytes, lymphocytes, and fibroblasts; it inhibits mitochondrial metabolism; it also inhibits nicotinamide adenine dinucleotide- (NADH-) dependent isocitrate dehydrogenase; and it decreases cyclic guanosine monophosphate (c-GMP) to normal in keratinocytes. It is available as ointment, cream, or gel (0.1–5%); a novel dithranol-containing lecithinized microemulsion systems with mean particle diameter of 72.8 nm composed of isopropyl myristate acetate and polyoxyethylene sorbitan oleate has shown enhanced skin permeation (82.23%), skin permeation flux (0.281 mg/cm^2^/h) along with skin retention (8.31%) and improves topical delivery of dithranol [89].

Anthralin is an effective therapeutic option for limited plaque psoriasis and AA; the advantage of the initial treatment is rapid penetration throughout the epidermis in less than 100 minutes; liposomal aqueous gel-based formulation causes only minimal coloration without irritation [90].

### 5.6. Zinc

Topical zinc sulphate cream 2.5% shows anti-inflammatory effects by inhibiting release of inflammatory cytokines IL-1 and IL-6 from keratinocytes; combined with 0.05% clobetasol cream (Zincoderm® cream) it showed good therapeutic effects in the treatment of chronic eczema, eczematous psoriasis, and lichen planus [91]. In eczematous forms of psoriasis, tests related to the mechanisms of development are directed to the investigation of contact sensitization [92]. The formulation with erythromycin (1.2% zinc + 4% erythromycin: Zineryt® solution), besides inhibition of IL-6 release from keratinocytes, lowers 5-alpha reductase in the sebaceous glands, which provides a beneficial effect in the treatment of acne [93].

### 5.7. Interferon Alpha

Topical IFN*α*2b drops (10^6^ IU/mL) were used 4 times per day for four months and demonstrated significant immunomodulating and antiallergic (antianaphylactic) effects in the treatment of vernal keratoconjunctivitis by directly inhibiting eosinophil differentiation and proliferation; blocking IgE-mediated histamine release in the tissues; limiting inflammation by inhibiting the release of IL-4 and IL-5 from Th2 cells; inhibiting the release of eosinophilic cationic protein, which plays an important role in the development of corneal damage; inhibiting the release of IL-10 from monocytes; and inhibiting overexpressed vascular endothelial growth factor (VEGF) [94]. When applied 4 times a day for 2 months, it showed significant immunomodulatory effects including antiviral, antiproliferative, and angiogenic activities in the treatment of conjunctival and corneal intraepithelial neoplasia [95].

## 6. Conclusion

Regulatory mechanisms of the immune response are numerous; it has been shown that topical immunomodulators interfere in all spheres: from anaphylaxis, eczema, and transplant to tumor immune responses. Once discovered, they have certainly proved their clinical effects, whereas their significance is measured by their therapeutic efficacy. The importance of topical immunomodulators is even greater bearing in mind that the skin is a mirror, and adjacent visible mucous membranes are the window to the human insight.

## Abbreviations

IL-2: Interleukin-2
IFN-*γ*: Interferon-*γ*
TCIs: Topical calcineurin inhibitors
TNF-*α*: Tumor necrosis factor-*α*
GM-CSF: Granulocyte-macrophage colony-stimulating factor
Fc*ε*RI: High-affinity receptors for immunoglobulin class E
LC: Langerhans cells
IDEC: Inflammatory dendritic epidermal cells
CCR7: Chemokine receptor 7
IgE: Immunoglobulin E
FDA: Food and Drug Agency
GVHD: Graft versus host disease
IGA: Investigator's Global Assessment
SLE: Systemic lupus erythematosus
CAD: Chronic actinic dermatitis
AD: Atopic dermatitis
T-MNLC: Modified nanolipid carrier system for topical delivery of tacrolimus
PG: Pyoderma gangrenosum
PGL: Localized pyoderma gangrenosum
DC: Dendritic cells
DTH: Delayed-type hypersensitivity reaction
AS: Allergic asthma
AR: Allergic rhinitis
AC: Allergic conjunctivitis
CS: Corticosteroids
PMNL: Polymorphonuclear leukocytes
RA: Rheumatoid arthritis
TSLP: Thymic stromal lymphopoietin
FKBP12: Macrophilin-12
BCC: Basal cell carcinoma
SCC: Squamous cell carcinoma
mTOR: Mammalian target of rapamycin
TSC: Tuberous sclerosis complex
DPCP: Diphenylcyclopropenone
SADBE: Squaric acid dibutyl ester
AA: Alopecia areata
HLA: Human leukocyte antigen
TGF-*β*: Transforming growth factor *β*
VEGF: Vascular endothelial growth factor
DNCB: Dinitrochlorobenzene
NMSC: Nonmelanoma skin cancers
HIV: Human immunodeficiency virus
HSV-2: Herpes simplex virus-type 2
VDR: Vitamin D receptors
NB-UVB: Narrow-band ultraviolet B
PUVA: Psoralen and ultraviolet A radiation
Cys A: Cyclosporin A
NADH: Nicotinamide adenine dinucleotide
c-GMP: Cyclic guanosine monophosphate.
